# Consumo crónico de edulcorantes en ratones y su efecto sobre el sistema inmunitario y la microbiota del intestino delgado

**DOI:** 10.7705/biomedica.5806

**Published:** 2021-09-22

**Authors:** Jorge Alberto Escoto, Beatriz Elina Martínez-Carrillo, Ninfa Ramírez-Durán, Hugo Ramírez-Saad, José Félix Aguirre-Garrido, Roxana Valdés-Ramos

**Affiliations:** 1 Investigación en Nutrición, Facultad de Medicina, Universidad Autónoma del Estado de México, Toluca, Estado de México, México Universidad Autónoma del Estado de México Facultad de Medicina Universidad Autónoma del Estado de México TolucaEstado de México Mexico; 2 Microbiología Médica y Ambiental, Facultad de Medicina, Universidad Autónoma del Estado de México, Toluca, Estado de México, México Universidad Autónoma del Estado de México Facultad de Medicina Universidad Autónoma del Estado de México TolucaEstado de México Mexico; 3 Departamento de Sistemas Biológicos, Universidad Autónoma Metropolitana-Xochimilco, Ciudad de México, México Universidad Autónoma Metropolitana Universidad Autónoma Metropolitana XochimilcoMéxico Mexico; 4 Biotecnología Ambiental, Universidad Autónoma Metropolitana-Lerma, Lerma, Estado de México, México Universidad Autónoma Metropolitana Universidad Autónoma Metropolitana LermaLerma Mexico

**Keywords:** edulcorantes, intestino delgado, microbioma gastrointestinal, sacarosa, estevia, Sweetening agents, intestine, small, gastrointestinal microbiome, sucrose, stevia

## Abstract

**Introducción.:**

Los edulcorantes son aditivos que se consumen en los alimentos. Pueden ser naturales (sacarosa y estevia) o artificiales (sucralosa). Actualmente, se consumen rutinariamente en múltiples productos, y sus efectos en la mucosa y la microbiota del intestino delgado aún son controversiales

**Objetivo.:**

Relacionar el consumo de edulcorantes y su efecto en el sistema inmunitario y la microbiota del intestino delgado en ratones CD1.

**Materiales y métodos.:**

Se utilizaron 54 ratones CD1 de tres semanas de edad divididos en tres grupos: un grupo de tres semanas sin tratamiento, un grupo tratado durante seis semanas y un grupo tratado durante 12 semanas. Se les administró sacarosa, sucralosa y estevia. A partir del intestino delgado, se obtuvieron linfocitos B CD19^+^ y células IgA^+^, TGF-β *(Transforming Growth Factor-beta)* o el factor de crecimiento transformador beta (TGF-beta), IL-12 e IL-17 de las placas de Peyer y de la lámina propia. De los sólidos intestinales se obtuvo el ADN para identificar las especies bacterianas.

**Resultados.:**

Después del consumo de sacarosa y sucralosa durante 12 semanas, se redujeron las comunidades bacterianas, la IgA+ y el TGF-beta, se aumentó el CD19+, y además, se incrementaron la IL-12 y la IL-17 en las placas de Peyer; en la lámina propia, aumentaron todos estos valores. En cambio, con la estevia mejoraron la diversidad bacteriana y el porcentaje de linfocitos CD19+, y hubo poco incremento de IgA+, TGF- β e IL-17, pero con disminución de la IL-17.

**Conclusión.:**

La sacarosa y la sucralosa alteraron negativamente la diversidad bacteriana y los parámetros inmunitarios después de 12 semanas, en contraste con la estevia que resultó benéfica para la mucosa intestinal.

## Abstract (en)

**Introduction:**

Sweeteners are additives used in different foods. They can be natural (sucrose and stevia) or artificial (sucralose). Currently, they are routinely consumed in multiple products and their effects on the mucosa of the small intestine and its microbiota are still controversial.

**Objective:**

To relate the consumption of sweeteners and their effect on the immune system and the microbiota of the small intestine in CD1 mice.

**Materials and methods:**

We used 54 three-week-old CD1 mice divided into three groups in the experiments: 1) A group of three weeks without treatment, 2) a group treated for six weeks, and 3) a group treated for 12 weeks using sucrose, sucralose, and stevia. We obtained CD19+ B lymphocytes, IgA+ antibodies, *transforming growth* factor-beta (TGF-b), and interleukins 12 and 17 (IL-12 and -17) from Peyer's patches and lamina propria cells while DNA was obtained from intestinal solids to identify bacterial species.

**Results:**

After 12 weeks, sucrose and sucralose consumption caused a reduction in bacterial communities with an increase in CD19+, a decrease in IgA+ and TGF-b, and an increase in IL-12 and -17 in the Peyer's patches while in the lamina propria there was an increase in all parameters. In contrast, stevia led to an improvement in bacterial diversity and percentage of CD19+ lymphocytes with minimal increase in IgA+, TGF-b, and IL-12, and a decrease in IL-17.

**Conclusion:**

Sucrose and sucralose caused negative alterations in bacterial diversity and immune parameters after 12 weeks; in contrast, stevia was beneficial for the intestinal mucosa.

Los edulcorantes son aditivos alimentarios que brindan un sabor dulce a los alimentos ^1^. Se clasifican en naturales (sacarosa y estevia) o artificiales (sucralosa) y, con base en su aporte energético, como nutritivos (sacarosa) o no nutritivos (sucralosa y estevia) ^2^. En los últimos años, se ha incrementado el uso de edulcorantes no nutritivos como alternativa para reducir el consumo de calorías y, así, combatir la elevada tasa de enfermedades crónicas no transmisibles, como obesidad, diabetes mellitus e hipertensión arterial sistémica ^3^. El metabolismo de los edulcorantes no nutritivos es independiente de la insulina y estos permiten disfrutar del sabor dulce en los alimentos sin incrementar el contenido calórico de la dieta ^4^. Los edulcorantes con mayor demanda comercial actualmente son sacarosa, sucralosa y estevia ^5^.

El encargado de absorber y metabolizar los nutrientes que ingresan al sistema digestivo es el intestino delgado, el cual los recibe ya digeridos por el estómago, los absorbe y los utiliza para proveer de energía al organismo ^6^. El intestino delgado está involucrado en la defensa del organismo mediante la reacción inmunitaria local ^7^^)^ del tejido linfoide asociado al intestino de tipo encapsulado (placas de Peyer) y el difuso (lámina propia) ^6^. Este tejido está separado de la luz intestinal por las células epiteliales ^8^, las cuales forman una barrera protectora y son el medio por el cual interactúan la microbiota y las placas de Peyer ^9^, que permiten el transporte de macromoléculas y microorganismos intactos que interaccionan con células del sistema inmunitario en ese sitio. La lámina propia, por su parte, recibe células activadas que han tenido contacto con los antígenos en las placas de Peyer ^10^.

Es evidente, entonces, la relación íntima entre el contenido intestinal, la microbiota y las estructuras linfoides del intestino delgado ^11^, cuyo funcionamiento y homeostasis dependen de su adecuado equilibrio, el cual se modifica según los nutrientes que se consumen y que alteran, a su vez, el tipo de microbiota y, por lo tanto, la inmunidad en este sitio. Otro factor que interviene en el mantenimiento de dicho equilibrio es la adecuada producción de IgA+ ^12^. Se ha observado que su ausencia en ratones altera la microbiota intestinal ^13^, es decir, que la microbiota comensal y la IgA+ pueden regularse entre sí promoviendo un "mutualismo pacífico" entre huésped y anticuerpos ^12^. Asimismo, este equilibrio permite que los linfocitos B se activen y migren de las placas de Peyer hacia la lámina propia del intestino delgado, permitiendo el cambio de isotipo a células plasmáticas IgA+ ^14^, lo que confirma la relación estrecha y simbiótica entre la composición de la microbiota y el desarrollo de la inmunidad del organismo. En pocos estudios se aborda la composición de la microbiota del intestino delgado en humanos debido a su difícil acceso, por lo que la mayoría se enfoca en la descripción de la microbiota del intestino grueso y su relación con la inmunidad.

En el intestino delgado, sin embargo, hay una estrecha relación entre el tipo y la proporción de nutrientes, el desarrollo de la microbiota y el estímulo del sistema inmunitario de las mucosas, especialmente de las placas de Peyer. Esto se ve favorecido por la secreción de algunas citocinas, como el TGF-beta, que regula muchas de las funciones celulares en el intestino ^15^, además de estimular la diferenciación de los linfocitos vírgenes a Th17, con la consecuente secreción de IL-17 ^16^^,^^17^. Esta interleucina está implicada en la defensa frente a infecciones bacterianas y fúngicas que no están cubiertas por la respuesta de los linfoitos T ayudadores 1 y 2 (Th1 y Th2) ^18^. Como se ha demostrado, las citocinas tienen la capacidad de distinguir la microbiota normal o natural de la patógena. Otro ejemplo de ello es la IL-12, la cual contribuye a resolver enfermedades infecciosas causadas por microorganismos patógenos, como *Listeria monocytogenes, Mycobacterium tuberculosis, Candida albicans* y *Leishmania major*^19^, aunque su actividad en el intestino delgado aún no es clara.

Todavía son pocos los estudios sobre la microbiota del intestino delgado y, más escasos aún, aquellos que explican la influencia del consumo de edulcorantes. Nuestro grupo ha llevado a cabo estudios previos sobre el consumo de edulcorantes en ratones jóvenes recién destetados ^20^^,^^21^, para relacionar su efecto con parámetros inmunitarios mediante diferentes métodos de análisis de la microbiota del intestino delgado.

En este contexto, el presente estudio se planteó como objetivo relacionar el consumo de edulcorantes y su efecto, sobre el sistema inmunitario y la microbiota del intestino delgado en ratones CD1.

## Material y métodos

### Diseño de estudio

Se hizo un estudio experimental, controlado y transversal con 54 ratones machos de tres semanas de edad (recién destetados) de la cepa CD1, libres de patógenos, alimentados con croquetas Rodent Laboratory Chow™ 5001 de Purina (3,02 kcal/g) (RLChow™ 5001, Saint Louis, MO, USA) y agua *ad libitum.* Los ratones fueron alojados en jaulas en grupos de cuatro en condiciones controladas de temperatura (19 a 21 ^o^C) y ciclos de luz-oscuridad de 12 horas cada uno. Se cuantificó el consumo de agua y alimento por semana hasta que concluyó el experimento.

El proyecto fue aprobado por el Comité de ética en investigación de la Facultad de Medicina de la Universidad Autónoma del Estado de México, y se consideraron las especificaciones técnicas para la producción, el cuidado y el uso de animales de laboratorio de la Norma Oficial Mexicana, NOM-062-ZOO-1999 22.

### Grupos de estudio

**Figura 1:**
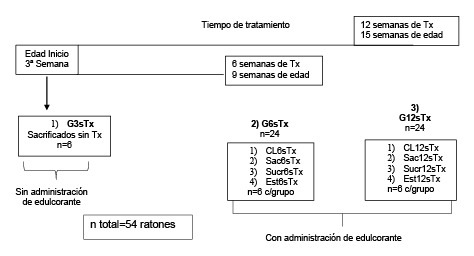
Distribución de los grupos de estudio de acuerdo con el tiempo de tratamiento y el tipo de edulcorante administrado

Se conformaron tres grupos de ratones utilizando como variables el tiempo de tratamiento (6 y 12 semanas) y el consumo de edulcorante administrado (figura 1): 1) un grupo de tres semanas de edad sin tratamiento (G3sSTx, n=6); los animales se sacrificaron el día 21 del destete y sirvieron de control inicial; 2) un grupo de tres semanas de edad y seis semanas de tratamiento (G6sTx, n=24) con edulcorantes a partir de la tercera y hasta la novena semana de vida de los ratones, y 3) un grupo de tres semanas de edad con 12 semanas de tratamiento (G12sTx, n=24) y administración de dos edulcorantes de la tercera a la decimoquinta semana de vida de los ratones. Los ratones del grupo G3sSTx fueron sacrificados recién destetados, al final de la tercera semana de vida, y no se les administró ningún tratamiento. Los grupos G6sTx y G12sTx fueron subdivididos en cuatro subgrupos (n=6 cada uno), según el tipo de edulcorante administrado (figura 1):

G6sTx:

- de control (CL6sTx), sin administración de edulcorante;
- grupo suplementado con sacarosa (Sac6sTx); iii) grupo suplementado con sucralosa (Sucr6sTx), y iv) grupo suplementado con estevia (Est6sTx).

G12sTx:

- de control (CL12sTx), sin administración de edulcorante;
- suplementado con sacarosa (Sac12sTx);
- grupo suplementado con sucralosa y
- grupo suplementado con estevia (Est12sTx).

### Administración de edulcorantes

Los edulcorantes diluidos en agua ultrapura según las recomendaciones de la Norma Oficial Mexicana NOM-218-SSA1-2011 para bebidas saborizadas no alcohólicas, se administraron por vía oral a libre demanda ^23^. La solución con el edulcorante se preparó en una concentración de 41,66 mg/ mi de sacarosa, y 4,16 mg/ml de sucralosa y estevia. Se colocó diariamente la solución con edulcorante en las jaulas de los ratones durante cinco horas en un horario de las 08:00 a las 13:00 pm. Después de las 13:00 horas, se retiraba la solución y se sustituía con agua sin edulcorante (de 13:00 p.m. a 08:00 a.m.). Se cuantificó diariamente el volumen de agua con edulcorante para determinar su consumo en mg/ml, así como el volumen de agua sin edulcorante para obtener la media de consumo por semana.

### Determinación del índice de masa corporal

Para determinar el índice de masa corporal (IMC), se cuantificaron el peso y la longitud (nariz-ano) de los animales semanalmente a partir de la semana 3 y hasta el momento del sacrificio, con ayuda de una báscula pesa ratón Triple Beam 700/800 Series (Ohaus™ Cat. No. 2,729,439). La longitud se determinó de la nariz hasta el ano en los ratones anestesiados con 0,1 ml de pentobarbital sódico al 1 %, usando una cinta métrica ^24^.

### Cálculo energético y consumo de alimento

Se cuantificó el consumo de alimento de forma semanal a partir de la tercera semana de vida de los animales, pesando el alimento colocado en las jaulas los días lunes (inicio) y domingo (final) para, así, calcular el consumo como la diferencia de cantidad al inicio y final de cada semana. El consumo energético total se calculó a partir del aporte energético del alimento Rodent Laboratory Chow™ 5001 de Purina (3,02 kcal/g) y de cada edulcorante: sacarosa, 4 kcal/gramo, sucralosa, 0 kcal/gramo, y estevia, 4 kcal/gramo. Una vez obtenidos los datos de consumo de alimento y agua con edulcorante por semana, se calculó la ingestión energética total con la fórmula: kcal = (gramos de alimento x 3,02) + kcal de edulcorante ^24^^,^^25^.

### Determinación de glucosa

Semanalmente, se determinó la glucosa en sangre periférica tomada de la cola del ratón por capilaridad con un glucómetro Contour TS™ de Bayer ^26^.

### Obtención y procesamiento de muestras

Los grupos de ratones fueron sacrificados a la tercera, novena y decimoquinta semanas de vida, en condiciones de ayuno de 8 horas. El procedimiento se realizó a las 07:00 a.m. por dislocación cervical, siguiendo las recomendaciones de la norma oficial mexicana para uso y manejo de animales de laboratorio (NOM-062-ZOO-1999) y después de anestesiarlos con 0,1 ml de pentobarbital sódico al 1 %. Una vez sacrificados, se procedió a disecar y lavar el intestino delgado cuidadosamente con 3 ml de solución tampón fosfato-salino (PBS) para eliminar el mesenterio y después extraer las placas de Peyer ^27^^,^^28^. El líquido obtenido se centrifugó a 4.000 rpm/5 minutos, se recolectó el contenido sólido de heces fecales y se guardó a -70 ° hasta su procesamiento. Para obtener los linfocitos de las placas de Peyer y la lámina propia, se utilizó la técnica descrita por Reséndiz-Albor, *et al.*^28^, con algunas modificaciones, como se describe a continuación.

### Aislamiento de linfocitos del intestino delgado (placas de Peyer y lámina propia)

*Placas de Peyer.* Una vez obtenidas, se trituraron en una solución de suero de ternera fetal (STF) y solución tampón (PBS) al 3 % en hielo, y se pasaron por un filtro de acero inoxidable de 300 secciones para células. El líquido se centrifugó durante 10 minutos a 1.500 rpm y 4° C, para obtener el botón de células.

*Lámina propia.* Para la recuperar los linfocitos de la lámina propia del intestino delgado, este se evertió introduciendo una aguja de ganchillo de hierro de 10 cm de largo atada a una cuerda. Se ató el intestino en un extremo, se retiró la aguja de ganchillo y se tiró de la cuerda con cuidado mientras este se mantenía sumergido en medio RPMI-1640 frío (Sigma-Aldrich, No. Cat. R65806, Saint Louis, MO, USA). Cada segmento intestinal evertido se transfirió a un tubo de 50 ml que contenía 25 ml de medio RPMI con 60 U/ml de colagenasa de tipo IV (Sigma-Aldrich, Cat. No. C5138, Saint Louis, MO, USA), DTT (1,4 Ditiotreitol, Sigma-Aldrich, Cat. No. 43819, Saint Louis, MO, USA), 1 % de SFT y 50 μg/ml de gentamicina.

Los tubos se incubaron horizontalmente durante 30 minutos a 37 °C en un baño de agua con agitación a 150 rpm. Los contenidos de cada tubo se transfirieron luego a placas de Petri y se agregaron 200 μl de SFT. La mucosa intestinal se comprimió con un émbolo de jeringa sobre una malla de plástico. La suspensión de cada tubo con células de lámina propia se filtró con una malla de organza y, después, se centrifugó durante 10 minutos a 1.500 rpm a 4° C. Las suspensiones celulares se recolectaron y centrifugaron en un gradiente discontinuo de 40 a 70 % de Percoll a 2.500 rpm durante 25 minutos. Las células de la interfaz se lavaron y se suspendieron en medio RPMI-1640.

### Citometría de flujo

La suspensión de células obtenida a partir de las placas de Peyer y la lámina propia se ajustó a 1 x10^6^ células/ml en solución tampón (PBS) para el análisis citofluorométrico, utilizando la técnica de Arciniega-Martínez 29 con algunas modificaciones:

- el fenotipo de superficie de las células B fue detectado utilizando un anticuerpo monoclonal marcado con fluorescencia anti-CD19 (PE, Cat. No. 553786, BD Biosciences, New Jersey, USA); las células se incubaron durante 30 minutos a temperatura ambiente, se lavaron con (PBS) y se fijaron con paraformaldehído;
- el porcentaje de células plasmáticas IgA+ se detectó agregando un coctel compuesto por anticuerpos anti-CD19 PE, anti-CD138 APC y anti-IgA FITC (todos de BD Biosciences, New Jersey, USA). Las células plasmáticas y las células B se fijaron, permeabilizaron y tiñeron, siguiendo el protocolo de BD Bioscience para tinción intracelular;
- para el reconocimiento de la producción de citocinas intracelulares, los linfocitos se estimularon con una mezcla que contenía miristato de forbol acetato, ionomicina y brefeldin A (Leucocyte Activation Cocktail Kit, BD Pharmingen, San José California, USA), y se incubaron durante 4 horas a 37 °C y 5 % de CO2.

Después, se añadieron anticuerpos contra los marcadores de superficie celular PerCP anti-CD4 y se incubaron como antes. Para la marca intracelular de células T CD4+, la fijación y la permeabilización se hicieron utilizando Cytofix/Cytoperm Kits (BD Pharmingen, San José California, USA) y siguiendo las instrucciones del fabricante. Estas células se incubaron con anti-IL-17 APC (Cat. No. 565163), anti-TGF-beta FITC (Cat. No. 141414) y anti-IL-12 PerCP (Cat. No. 501821). La intensidad de la señal fluorescente se registró y analizó mediante un citómetro de flujo FACS Aria (Becton Dickinson). Los eventos se recogieron de la ventana de linfocitos en el diagrama de puntos FSC/SSC. Se adquirieron 20.000 eventos cerrados de cada muestra utilizando el programa de investigación CellQuest (Becton Dickinson). Los datos se analizaron con el programa Summit, versión 4,3 (Dako, Colorado Inc.). Se reportaron los datos de seis ratones por grupo como medias ± desviación estándar (DE).

### Extracción y secuenciación del ADN

A partir de los sólidos intestinales obtenidos de cada ratón, se extrajo el ADN metagenómico y se cuantificó por medio de electroforesis en gel de agarosa al 0,8 %, utilizando como marcador de tamaño molecular el Phago Lambda DNA/EcoRI y el programa Kodak Electrophoresis Documentation and Analysis System (EDAS) 290. Para explorar la presencia de genes o familias génicas en la microbiota intestinal de los ratones de cada grupo de tratamiento, se hizo una ultrasecuenciación directa en la plataforma ILLUMINA del ADN metagenómico de muestras seleccionadas con base en los datos de diversidad y composición diferencial. El ADN metagenómico se envió al servicio de secuenciación masiva *Integrated Microbiome Resource* (Canadá). La secuenciación de la región V6-V8 del gene *16S ARNr* se hizo en la plataforma MiSeq de Illumina.

### Análisis y clasificación de secuencias metagenómicas

El análisis bioinformático se hizo con el programa MOTHUR ^30^, así como el ensamble de las secuencias Read1 y Read2; para tener una secuencia aproximada de 450 pb, se verificó que las secuencias cumplieran con los siguientes criterios: no presentar bases ambiguas, tener una longitud mínima de 400 pb, no contener homopolímeros mayores de 8 pb, y tener una ampliación de secuencias de calidad de 50 pb y una puntuación media por secuencia de 35 pb.

Las unidades taxonómicas operacionales *(Operational Taxonomic Unit,* OTU) se asignaron utilizando la base de datos Silva, versión 119 ^31^; el alineamiento de referencia bacteriana se hizo con las secuencias que presentaran un 97 % de similitud y, además, se identificaron y excluyeron las secuencias quiméricas con el programa UCHIME ^32^^,^^33^.

La diversidad y riqueza de las especies presentes en las muestras metagenómicas, así como las curvas de rarefacción, se calcularon con un 97 % de similitud como parte de los resultados de diversidad alfa obtenidos a partir del análisis en el programa MOTHUR.

Se utilizaron cinco indicadores para determinar la evolución de las comunidades bacterianas, como los índices de diversidad de Shannon y Pielou, y los estimadores Inv-Simpson y Chao1 para la riqueza de especies, calculados con base en el número de unidades taxonómicas operacionales observadas ^34^. Estos parámetros se calcularon mediante la normalización de todas las bibliotecas con base en la muestra que presentó el menor número de secuencias.

El análisis de componente principal y el análisis estadístico multivariado se hicieron utilizando el programa STAMP, con una agrupación de 97 % de identidad ^35^. Las secuencias obtenidas se depositaron en la base de datos del *National Center for Biotechnology Information* (NCBI) con el número de acceso SRP149731.

### Análisis estadístico

Para los datos morfométricos, nutricionales e inmunológicos, se obtuvieron medidas de tendencia central, medias y desviación estándar; se utilizó la prueba de Kologorov-Smirnov para comprobar la normalidad de los datos con un valor de significación de p<0,05.

Una vez comprobada la normalidad de los datos, se procedió a aplicar la prueba de análisis de varianza ANOVA de una vía para comparar los grupos según el tipo de tratamiento (control, sacarosa, sucralosa y estevia) a las 6 y 12 semanas de tratamiento, y a una ANOVA de dos vías para comparar los grupos a la tercera, sexta y duodécima semana por tiempo de consumo y tipo de edulcorante.

En ambos casos, se hizo la prueba *post hoc* de Bonferroni con una significación estadística de p<0,05. Los datos obtenidos del análisis de secuencias metagenómicas mediante MOTHUR, se analizaron y graficaron en el programa STAMP, versión 2,3, utilizando un ANOVA de una vía y la prueba *post hoc* de Tukey-Kramer.

## Resultados

### Consumo de agua y alimento, aporte energético e IMC

*El consumo de agua fue menor en el subgrupo Sac12sTx del G12sTx.* En el G3sSTx, se cuantificaron el consumo de agua y alimento, el aporte calórico y el IMC después del destete de los animales, los cuales fueron sacrificados inmediatamente. En el G6sTx, no se apreciaron diferencias en el consumo de agua (p=0,635), pero en el G12sTx este disminuyó en el subgrupo Sac12sTx (p<0,042). Al comparar los grupos por tiempo de tratamiento, se encontraron diferencias significativas (p<0,042) entre el G3sSTx y el G12sTx, con un incremento en el consumo de agua (cuadro 1).

**Cuadro 1:** Composición de la dieta normal administrada a los ratones durante 6 y 12 semanas

Porcentaje	
Macronutrientes	Contenido de la dieta normal (%)
Hidratos de carbono	4B,7
Lípidos	10,7
Proteínas	23,9
Micronutrientes	
Vitaminas	4,6
Minerales	7,0
Fibra	5,1
Total	100
Aporte energético (kcal/g)	3,02

Contenido de la dieta normal: Rodent Laboratory Chow™ 5001 de Purina administrada a los ratones. El porcentaje total de nutrientes digeribles fue de 76 % y la dieta aportó 3,02 kcal/g. Se administraron los edulcorantes a libre demanda durante 6 y 12 semanas en una concentración de 41,66 mg/ml de sacarosa y 4,16 mg/ml de sucralosa y estevia, en el horario de las 08 a las 13 horas diariamente (lunes a domingo).

*El consumo de alimento disminuyó en los subgrupos de sacarosa a las 6 y 12 semanas de tratamiento.* En el G6sTx, el consumo de alimento disminuyó en los subgrupos Sac6sTx y Est6sTx (p<0,025); en el G12sTx, se observó disminución en los subgrupos Sac12sTx y Sucr12sTx (p<0,007) comparados con sus respectivos grupos de control. La diferencia entre los grupos según el tiempo de tratamiento fue significativa (p<0,001) (Verificar sentido con los autores). El consumo crónico de edulcorantes redujo el consumo de alimento, según se comprobó al comparar los resultados de los subgrupos CL6sTx y Sac6sTx a las 6 (p<0,011) y 12 semanas (p<0,020) de tratamiento (cuadro 2).

**Cuadro 2:** Parámetros morfométricos y nutricionales de los grupos que consumieron edulcorantes durante 6 y 12 semanas

IMC (g/cm2) Consumo de agua (ml) ** Glucemia (mg/l)	G3sSTx				
	Media ± DE				
	n=6				
	0,192 ± 0,2				
	4,4 ± 0,194				
	95 ± 8,45				
	G6sTx CL6sTx	Sac6sTx	Sucr6sTx	Est6sTx	
	Media ± DE	Media ± DE	Media ± DE	Media ± DE	
	n=6	n=6	n=6	n=6	P*
IMC (g/cm2)	0,294 ± 0,02	0,286 ± 0,013	0,298 ± 0,015	0,3 ± 0,016	0,408
Consumo de agua (ml) ^**^	8,3 ± 0,839	7,8 ± 0,248	7,9 ± 0,019	8,2 ± 0,276	0,635
Consumo de alimento (g) ^**^	39,2 ± 2,37	37,4 ± 0,427^a^	38,5 ± 0,066	37,3 ± 1,36 ^a^	0,025*
Consumo de edulcorante ^**^ (mg/ml)	0	128 ± 9,79^b^	22,7 ± 0,4	14,3 ± 1,22	0,001*
Aporte energético (kcal) ^**^	118 ± 7,18	113 ± 1,29^a^	116 ± 0,193	112 ± 4,11 ^a^	0,028*
Glucemia (mg/dl)	135 ± 12	133 ± 2,7	128 ± 9	142 ± 9	0,689
	G12sTx				
	CL12sTx	Sac12sTx	Sucr12sTx	Est12sTx	
IMC (g/cm2)	0,275 ± 0,013	0,288 ± 0,02	0,289 ± 0,014	0,289 ± 0,017	0,293
Consumo de agua (ml) ^**^	8,1 ± 1,14	7,3 ± 0,267^a^	7,7 ± 0,419	8,2 ± 0,229	0,042*
Consumo de alimento (g) ^**^	35,1 ± 2,87	31,3 ± 1,45^a^	33,6 ± 1,06 a	34,8 ± 2,71	0,007*
Consumo de edulcorante ^**^ (mg/ml)	0	200 ± 6,68^b^	28,4 ± 0,6	29 ± 0,644	0,001*
Aporte energético (kcal) ^**^	106 ± 8,68	94,9 ± 4,39^a^	101 ± 3,22	105 ± 8,18	0,008*
Glucemia (mg/dl)	136 ± 10	134 ± 14	130 ± 8,9	140 ± 3,2	0,648

*ANOVA de una vía para comparar los subgrupos por tipo de edulcorante consumido: ^a^ prueba post-hoc de Bonferroni, con p<0,05, de los subgrupos que observaron disminución en su valor comparados con su control. ^b^ prueba post-hoc de Bonferroni, con p<0,05, de los que presentaron incremento en su valor comparados con su control. ** ANOVA de dos vías entre las variables 3sSTx, 6sTx y 12sTx con diferencias significativas (p<0,05) comparados en cuanto a tiempo de exposición a los edulcorantes

Los valores representan la media ± desviación estándar de los valores morfométricos y nutricionales de los ratones que consumieron edulcorantes durante 6 y 12 semanas. Los experimentos se realizaron por duplicado. Grupo de tres semanas sin tratamiento (3sSTx); grupos con 6 semanas de tratamiento (G6sTx): control (CL6sTx), sacarosa (Sac6sTx), sucralosa (Sucr6sTx) y estevia (Est6sTx); grupos con 12 semanas de tratamiento (G12sTx): control (CL12sTx), sacarosa (Sac12sTx), sucralosa (Sucr12sTx) y estevia (Est12sTx). IMC: índice de masa corporal

*El aporte energético se encontró reducido en los subgrupos de edulcorantes.* En el G6sTx, el aporte energético se redujo en los subgrupos Est6sTx y Sac6sTx (p<0,049) comparados con su control. En el G12sTx, esta reducción se apreció entre los subgrupos CL12sTx y Sac12sTx (p<0,012) y Sac12sTx y Est12sTx (p<0,022). La diferencia entre los grupos fue significativa (p<0,001); el G6sTx tuvo mayor aporte energético comparado con el G12sTx, lo que significa que el tiempo de consumo sí modificó los hábitos de consumo tanto del edulcorante como del alimento (cuadro 2).

*El IMC no se modifcó con el consumo de edulcorantes.* La reducción en el consumo de alimento y de edulcorantes con bajo aporte energético pueden ser la causa de que el IMC no sufriera modificaciones significativas entre los grupos G6sTx (p<0,408) y G12sTx (p<0,293) (cuadro 2).

### Consumo de edulcorante y glucemia

*El edulcorante más consumido por los ratones fue la sacarosa.* Se comparó el consumo de edulcorantes excluyendo los subgrupos de control (no consumieron edulcorante) (cuadro 2). Los ratones consumieron la sacarosa en gran cantidad: 128 mg (6sTx) y 200 mg (12sTx), por lo cual las diferencias fueron significativas entre los subgrupos Sac6sTx y Sac12sTx (p<0,001), independientemente del tiempo de consumo en ambos grupos. Por el contrario, no se observaron diferencias entre los subgrupos de sucralosa y estevia (p=0,083) a las 6 ni a las 12 semanas de tratamiento (cuadro 2). Al comparar los grupos por tiempo, se encontró que, al final de las 12 semanas, se había consumido mayor cantidad de edulcorantes (p<0,001) en comparación con el G6sTx (cuadro 2).

*La glucemia no se modificó con el consumo de edulcorantes.* La glucemia de los grupos G6sTx y G12sTx se incrementó (p<0,001), comparada con la del G3sSTx. A pesar de este incremento, al comparar los grupos G6sTx y G12sTx, no se evidenció diferencia entre ellos (p=0,874), y la glucemia se mantuvo sin cambios según el tiempo y el consumo del edulcorante (cuadro 2).

### Marcadores inmunológicos en linfocitos de las placas de Peyer y la lámina propia del intestino delgado

En las placas de Peyer, el porcentaje de linfocitos CD19+ se incrementó paulatinamente con el consumo prolongado de edulcorantes en el grupo G12sTx. En el G6sTx, este aumento fue significativo en los subgrupos Suc6sTx y Est6sTx (p<0,001) comparados con el subgrupo CL6sTx. En el grupo G12sTx, los subgrupos Sac12sTx, Sucr12sTx y Est12sTx registraron aumento del porcentaje de linfocitos B CD19+ (p<0,001). Al comparar los grupos G3sSTx, G6sTx y G12sTx, se observó un incremento significativo (p<0,001) del porcentaje de linfocitos CD19+ después de 12 semanas de tratamiento (cuadro 3).

**Cuadro 3:** Porcentaje de linfocitos obtenidos de las placas de Peyer y la lámina propia del intestino delgado de ratones CD1 con consumo de edulcorantes por 6 y 12 semanas

	G3sSTx				
	Media ±DE				
	n=6				
CD19+PP (%)	55,1 ± 0,545				
IgA+ PP (%)	5,6 ± 0,267				
CD19+LP(%)	10,1 ± 0,577				
IgA+ LP (%)	16,3 ± 0,176				
	CL6sTx	Sac6sTx	Sucr6sTx	Est6sTx	
	Media ± DE n=6 (%)	Media ± DE n=6 (%)	Media ± DE n=6 (%)	Media ± DE n=6 (%)	P
CD19+PP (%) ^**^	55,7 ± 0,481	56,9 ± 0,513	64,8 ± 0,49^b^	59 ± 0,229^b^	0,001*
IgA+ PP (%)^**^	8,7 ± 0,32	17,7 ± 0,32^b^	17,1 ± 0,267^b^	10,7 ± 0,347^b^	0,001*
CD19+LP (%)**	8,07 ± 0,085	3,4 ± 1,19^a^	3,4 ± 1,09^a^	8,9 ± 0,759	0,001*
IgA+ LP (%) ^**^	20,7 ± 0,342	12,2 ± 0,261^a^	34,6 ± 0,55^b^	7,9 ± 0,545^a^	0,001*
	G12sTx				
	CL12sTx	Sac12sTx	Sucr12sTx	Est12sTx	
CD19+PP (%) ^**^	63,6 ± 0,63	71,9 ± 0,497^b^	69 ± 0,539^b^	65,1 ± 1,03^b^	0,001*
IgA+ PP (%) ^**^	11,3 ± 0,324	6,08 ± 0,267^a^	6,3 ± 0,32^a^	17,6 ± 0,267^b^	0,001*
CD19+LP (%) ^**^	3,4 ± 0,267	3,7 ± 0,176	4,8 ± 0,63^b^	9,1 ± 0,235^b^	0,001*
IgA+ LP (%) ^**^	16,3 ± 0,267	26,4 ± 0,545^b^	24,3 ± 0,267^b^	19,7 ± 0,347^b^	0,001*

*ANOVA de una vía para comparar los subgrupos por tipo de edulcorante consumido: ^a^ prueba post-hoc de Bonferroni, con p<0,05, de los subgrupos que disminuyeron el porcentaje de linfocitos y células IgA+ obtenidos de las placas de Peyer (PP) y la lámina propia (LP) al compararlos con su control. ^b^ Prueba post-hoc de Bonferroni, con p<0,05, que incrementaron el porcentaje de linfocitos y células IgA+ obtenidos de las placas de Peyer (PP) y la lámina propia (LP) comparados con su control. "Comparación de los grupos por tiempo de tratamiento con los edulcorantes (3sSTx, 6sTx y 12sTx) mediante ANOVA de dos vías y con diferencias significativas positivas (p<0,05).

Los valores representan la media ± desviación estándar del porcentaje de linfocitos CD19+ e IgA+ de las placas de Peyer y la lámina propia del intestino delgado, obtenidos por citometría de flujo con anticuerpos monoclonales. Los experimentos se realizaron por duplicado. Clusters de diferenciación (CD); inmunoglobulina A+ (IgA+); placas de Peyer (PP), lámina propia (LP). Grupo con tres semanas sin tratamiento (G3sSTx), grupos con 6 semanas de tratamiento (G6sTx): control (CL6sTx), sacarosa (Sac6sTx), sucralosa (Sucr6sTx) y estevia (Est6sTx); grupos con 12 semanas de tratamiento (G12sTx): control (CL12sTx), sacarosa (Sac12sTx), sucralosa (Sucr12sTx) y estevia (Est12sTx)

*El consumo de estevia incrementó el porcentaje de células IgA+ en los grupos G6sTx y G12sTx.* El porcentaje de células IgA+ en el grupo G6sTx se elevó con el consumo de Sac6sTx, Sucr6sTx y Est6sTx (p<0,001) comparado con el CL6sTx. Por el contrario, en el G12sTx, el porcentaje de IgA+ disminuyó en los subgrupos Sac12sTx y Sucr12sTx (p<0,001), pero se incrementó en el subgrupo Est12sTx (p<0,001) comparado con el grupo CL12sTx. El porcentaje de células IgA+ se elevó paulatina y significativamente en el G6sTx y el G12sTx (p<0,001), comparados con el G3sSTx (cuadro 3).

*Secreción de las citocinas TGF-beta, IL-12 e IL-17 en las placas de Peyer.* La concentración de TGF-beta en el G6sTx se incrementó de forma significativa (p<0,001) con el consumo de Sac6sTx, Sucr6sTx y Est6sTx. El comportamiento de la secreción del TGF-beta en el G12sTx fue diferente, pues en los subgrupos Sac12sTx y Sucr12sTx su secreción se deprimió significativamente (p<0,001), en tanto que se elevó en el subgrupo Est12sTx, comparados con el subgrupo CL12sTx. Al comparar los grupos, se observó un incremento de la secreción (p<0,001) del TGF-beta con el consumo de estevia por tiempo prolongado (cuadro 4).

**Cuadro 4:** Concentración intracelular de citocinas obtenidas de linfocitos de las placas de Peyer y la lámina propia de ratones CD1 sometidos a consumo de edulcorantes por 6 y 12 semanas

	G3sSTx				
	Media ± DE n=6				
TGF-β PP (pg/ml)	3,4 ± 0,053				
TGF-β PP (pg/ml) IL-12 PP (pg/ml)	3 ± 0,069				
IL-12 PP (pg/ml) IL-17 PP (pg/ml)	1,4 ± 0,058				
TGF-β LP (pg/ml)	10,8 ± 0,053				
IL-12 LP (pg/ml)	6,7 ± 0,122				
IL-17 LP (pg/ml)	2,8 ± 0,112				
	G6sTx				
	CL6sTx	Sac6sTx	Sucr6sTx	Est6sTx	
	Media ± DE n=6 (pg/ml)	Media ± DE n=6 (pg/ml)	Media ± DE n=6 (pg/ml)	Media ± DE n=6 (pg/ml)	Valor P
TGF-β PP (pg/ml) **	3,4 ± 0,053	11,6 ± 0,106b	12,2 ± 0,069b	11,2 ± 0,096b	0,001^*^
IL-12 PP (pg/ml) **	2,1 ± 0,09	2,2 ± 0,08	2,7 ± 0,069b	1,4 ±0,058a	0,001^*^
IL-17 PP (pg/ml) **	1,1 ± 0,08	2,9 ± 0,074b	3,8 ± 0,058b	3,3 ±0,074b	0,001^*^
TGF-β LP (pg/ml) **	11,5 ± 0,053	9,5 ±0,106	16,9 ± 0,069b	5,1 ± 0,096a	0,001^*^
IL-12 LP (pg/ml) **	5,4 ± 0,096	12,9 ±0,096b	8,1 ± 0,069b	2,8 ± 0,096a	0,001^*^
IL-17 LP (pg/ml) **	3,8 ± 0,074	7,4 ± 0,074b	7,8 ± 0,058b	11,1 ± 0,074b	0,001^*^
	G12sTx				
	CL12sTx	Sac12sTx	Sucr12sTx	Est12sTx	
TGF-β PP (pg/ml) ^**^	9,3 ± 0,16	1,8 ± 0,16a	1,97 ± 0,16a	18,96 ± 0,096b	0,001^*^
IL-12 PP (pg/ml) ^**^	2,3 ± 0,09	3,1 ±0,08b	1,08 ± 0,069a	7,26 ± 0,058b	0,001^*^
IL-17 PP (pg/ml) ^**^	1,7 ± 0,08	4,4 ±0,235b	5 ±0,053b	5,32 ± 0,074b	0,001^*^
TGF-β LP (pg/ml) ^**^	11,5 ± 0,053	17,8 ± 0,106b	18,7 ± 0,069b	16,6 ± 0,096b	0,001^*^
IL-12 LP (pg/ml) ^**^	4,5 ± 0,096	5,2 ± 0,096b	10,6 ±0,069b	1,2 ± 0,096a	0,001^*^
IL-17 LP (pg/ml) ^**^	3,3 ± 0,058	12,2 ± 0,074b	12 ± 0,058b	10,1± 0,08b	0,001^*^

*ANOVA de una via para comparar los subgrupos por tipo de edulcorante consumido: a prueba posthoc de Bonferroni, con p<0,05, de los subgrupos que disminuyeron la concentracion de citosinas comparados con su control; b prueba post-hoc de Bonferroni, con p<0,05, que incrementaron la concentracion de citocinas comparados con su control. **Comparacion entre los grupos por tiempo de exposicion al edulcorante (3sSTx, 6sTx y 12sTx), con ANOVA de dos vias y diferencias significativas positivas (p<0,05).

Los valores representan la media ± desviacion estandar de la concentracion intracelular de las citocinas (TGF-β, IL-12 e IL-17), obtenidas por citometria de flujo de linfocitos CD19+ de las placas de Peyer y la lamina propia. Los experimentos se realizaron por duplicado. Grupo de tres semanas sin tratamiento (G3sSTx); grupos con 6 semanas de tratamiento (G6sTx): control (CL6sTx), sacarosa (Sac6sTx), sucralosa (Sucr6sTx) y estevia (Est6sTx); grupos con 12 semanas de tratamiento (G12sTx): control (CL12sTx), sacarosa (Sac12sTx), sucralosa (Sucr12sTx) y estevia (Est12sTx). Placas de Peyer (PP); lamina propia (LP); factor de crecimiento transformador (TGF-☒); interleucinas 12 y 17 (IL-12, IL-17); picogramos por mililitro (pg/ml)

*El consumo prolongado de sucralosa redujo la secreción de IL-12 pero esta se incrementó con el consumo de estevia.* La secreción de IL-12 de los subgrupos CL6sTx y CL12sTx disminuyó con respecto al G3sSTx (cuadro 4). Al comparar los subgrupos del G6sTx, la concentración de IL-12 disminuyó significativamente en el subgrupo Est6sTx (p<0,001), y se incrementó con el consumo de Sucr6sTx comparado con el subgrupo CL6sTx (cuadro 4). En el G12sTx, la IL-12 se incrementó en los subgrupos Sac12sTx, especialmente en el Est12sTx, a diferencia del Sucr12sTx, en el que hubo una reducción en la secreción de IL-12 (p<0,001) (cuadro 3). Al comparar los grupos G3sStx, G6sTx y G12sTx, se apreció que, con el consumo de sacarosa y de estevia, se elevó la concentración de IL-12 (p<0,001).

*La secreción de IL-17 se incrementó con el consumo prolongado de edulcorantes.* La secreción de IL-17 se incrementó en los subgrupos Sac6sTx, Sucr6sTx y Est6sTx a las 6 semanas de tratamiento (p<0,001), comparados con el subgrupo CL6sTx. Con 12 semanas de tratamiento, la secreción de esta interleucina se encontró fue más abundante en todos los subgrupos que consumieron edulcorantes (cuadro 4). Al comparar los grupos, se apreció una diferencia paulatina y significativa (p<0,001) pues, cuanto mayor el tiempo de consumo de edulcorantes, mayor la concentración de la interleucina 17.

En la lámina propia, con el consumo de sacarosa y sucralosa (p<0,001), se disminuyó el porcentaje de linfocitos B CD19+ a las 6 semanas de tratamiento (G6sTx) y no hubo cambios en la secreción con el consumo de estevia (p=0,369). En el G12sTx, el incremento se observó en los subgrupos de estevia y sucralosa (p<0,001) (cuadro 3). Al comparar los grupos según el tiempo de tratamiento, se apreció una reducción del porcentaje de linfocitos (p<0,001) a las 6 (G6sTx) y 12 (G12sTx) semanas de tratamiento, comparados con el G3sTx (cuadro 3).

El consumo prolongado de edulcorantes modificó el porcentaje de IgA^+^ en la lámina propia. A las seis semanas de tratamiento, el porcentaje de IgA^+^ disminuyó con el consumo de Sac6sTx y Est6sTx (p<0,001) y se incrementó significativamente con la Sucr6sTx, comparado con el subgrupo CL6sTx. En cambio, a las 12 semanas de consumo, se elevó en todos los subgrupos el porcentaje de IgA+ (cuadro 3). Al comparar los grupos según el tiempo de exposición al edulcorante (6 y 12 semanas), se observaron diferencias (p<0,001) que disminuyeron con el consumo de sucralosa, pero se incrementaron con el de estevia comparados con sus controles, por lo cual la diferencia se puede atribuir al tipo de edulcorante y no al tiempo de exposición (cuadro 3).

Secreción de citocinas *TGF-* beta, *IL-12* e *IL-17* en la lámina propia. El TGF-beta en el G6sTx se encontró elevado en el subgrupo Sucr6sTx y disminuido en el subgrupo Est6sTx (p<0,001) al compararlos con el subgrupo CL6sTx. Sin embargo, en el G12sTx, se elevó en todos los subgrupos: Sucr12sTx, Sac12sTx y Est12sTx, en ese orden (cuadro 4). Al comparar los grupos por tiempo de tratamiento, no hubo diferencia entre el G3sSTx y los controles de los grupos G6sTx y G12sTx (p=0,873), pero sí entre los controles y los subgrupos de edulcorantes, por lo cual las diferencias dependieron del consumo del edulcorante y no del tiempo de exposición, como sucedió con la IgA^+^ (cuadros 3 y 4).

La secreción de *IL-12* se encontró elevada con el consumo de sacarosa y sucralosa, pero disminuyó con la estevia. En sincronía con lo anterior, la secreción de IL-12 en los G6sTx y G12sTx se elevó de forma significativa con el consumo de sacarosa y sucralosa (p<0,001), pero la secreción de IL-12 disminuyó en ambos grupos con el consumo de estevia (p<0,001). Al comparar los grupos por tiempo de tratamiento, se observó que la IL-2 se incrementó (p<0,001) a las 6 y 12 semanas de consumo de edulcorantes, comparados con el grupo G3sSTx (cuadro 4).

*La IL-17 se elevó en todos los subgrupos suplementados con edulcorantes a las 6 y 12 semanas.* En el G6sTx, la IL-17 se elevó con el consumo de los tres edulcorantes (cuadro 4). En el caso del G12sTx, el incremento fue similar en todos los subgrupos. Al comparar los grupos por tiempo de tratamiento, la IL-17 aumentó (p<0,001) en los grupos G6sTx y G12sTx, comparados con el G3sSTx.

### Diversidad bacteriana

La estructura de las comunidades bacterianas derivadas de la secuenciación del gen *16S* se abordaron mediante la normalización del número de secuencias en las bibliotecas antes del cálculo de los parámetros de cobertura, diversidad, riqueza y uniformidad de Good. En cuanto al primer parámetro (Good), se observó una alta proporción de unidades taxonómicas operacionales con relación a las esperadas (90-99 % de cobertura) en todas las librerías (cuadro 5). En las muestras del grupo G3sSTx, los grupos bacterianos con el porcentaje de abundancia relativa más alto correspondieron a *Pseudomonas, Lactobacillus* y *Clostridium* (cuadro 6).

**Cuadro 5:** Número de lecturas calificadas para la diversidad, riqueza y uniformidad del ADN de las comunidades bacterianas presentes en los sólidos intestinales de ratones CD1 sometidos a consumo crónico de edulcorantes

Grupo	Número de secuencias^a^	OTU^b^	Cobertura de Good^c^	Inv. Simpson^c^ (inferior/superior)	Chao1^c^ (inferior/superior)	H’^c^ (inferior/superior)	J’^c^
G3sSTx	58.350	270	0,99	1,965 (1,94/1,98)	1.691,1 (1.770,17/2.808,11)	0,75 (0,74/0,76)	0,93
CL6sTx	35.283	510	0,99	13,59 (13,28/13,92)	1.215,40 (1.006,39/1.524,73)	3,06 (3,04/3,08)	0,99
CL12sTx	36.357	340	0,99	5,09 (5,02/5,17)	1.090,6 (830,35/1.498,58)	2,24 (2,22/2,26)	0,99
SAC6sTx	28.979	849	0,97	7,084 (6,92/7,24)	2.893,9 (2.502,5/3.406,9)	2,67 (2,65/2,70)	0,99
SAC12sTx	72.818	232	0,99	1,06 (1,05/1,06)	1.106,5 (762,07/1.675,9)	0,159 (0,153/0,165)	0,95
SUC6sTx	33.594	216	0,99	15,63 (15,30/15,98)	1.525,58 (1.323,1/1.799,04)	2,92 (2,90/2,94)	0,98
SUC12sTx	51.654	727	0,98	1,29 (1,28/1,29)	1.268,3 (805,6/2.099,2)	0,45 (0,44/0,46)	0,97
EST6sTx	21.037	2.510	0,90	14,14 (13,77/14,54)	8.904,9 (8.030,3/9.918,35)	4,13 (4,10/4,17)	0,99
EST12sTx	24.650	281	0,99	7,01 (6,85/7,17)	760,7 (582,6/1.049,9)	2,52 (2,49/2,54)	0,99

* El número de secuencias se obtuvo después de eliminar las secuencias de baja calidad (N ≥2; homopolímeros ≥8) y las secuencias cortas (<250 nt).

^b^ El número de unidades taxonómicas operacionales (OTU) observadas se definió sobre la base de una distancia máxima del 3 %.

^c^ La biblioteca más pequeña (21.037 secuencias) se utilizó para la normalización de datos. Los resultados presentados son el promedio de 1.000 repeticiones. Los parámetros se estimaron utilizando MOTHUR. Los parámetros indican el número de lecturas calificadas, el número estimado de OTU y la cobertura, los estimadores de riqueza de especies (Good, Inv-Simpson), la diversidad de muestras (Chao 1), la diversidad (H') y la uniformidad (J') del ADN de las comunidades bacterianas presentes en los sólidos intestinales de ratones CD1 sometidos a consumo crónico de edulcorantes.

**Cuadro 6:** Abundancia relativa de los géneros bacterianos predominantes en las secuencias del gen de 16S rRNA de muestras intestinales sólidas de ratones CD1 sometidos al consumo de edulcorante por 6 y 12 semanas de tratamiento

Clase o filo	Género	Control %	Sacarosa %	Sucralosa %	Estev %
G3sSTx					
y-proteobacteria	*Pseoudomonas*	78,76	-	-	-
Firmicutes	*Lactobacillus*	2,25	-	-	-
	*Clostridium*	1,74	-	-	-
G6sTx					
α-proteobacteria	*Devosia* *Caulobacter* *Rhizobium*	- - -	1,63 1,60 1,08	- - -	6,20 5,80 4,21
β-proteobacteria	*Methylotenera* *Polaromonas* *Brevundimonas* *Albidiferax* *Massilia*	- - - - -	11,05 2,25 - - -	- - - - -	41,39 8,74 2,18 2,16 1,83
δ- proteobacteria	*Stigmatella*	-	-	-	1,23
ε-proteobacterias	*Helicobacter*	9,56	2,94	2,53	
y-proteobacteria	*Pseoudomonas*	-	6,13	16,50	8,94
Firmicutes	*Lactobacillus* *Clostridium* *Turicibacter* *Desulfosporosinus*	13,42 38,77 2,67 -	18,13 27,51 - -	20,30 36,80 1,45 -	- - - 2,79
Tenericutes	*Mycoplasma*	2,25	5,04	2,80	-
Actinobacteria	*Arthrobacter*	-	-	-	1,62
G12sTx					
ε -proteobacterias	*Helicobacter*	1,98	-	-	17,38
y-proteobacteria	*Pseudomonas*	-	99,1	96,46	1,17
Firmicutes	*Lactobacillus* *Clostridium* *Turicibacter* *Desulfosporosinus* *Streptococcus*	42,18 25,99 9,75 - -	- - - - -	2,33 - - - -	17,14 37,27 6,77 - 2,09
Tenericutes	*Mycoplasma*	6,24	-	-	10,08

En el cuadro se muestra el porcentaje (%) de la abundancia relativa de los géneros bacterianos predominantes según tiempo y tipo de tratamiento. Todos los géneros presentaron diferencias significativas y una abundancia relativa >1.

Las muestras de los subgrupos expuestos al consumo de edulcorantes CL6sTx, Sucr6sTx, Est6sTx y CL12sTx, presentaron una mayor riqueza según el índice de Shannon (H). Las muestras de los animales sometidos a consumo de edulcorante por 6 semanas (G6sTx), presentaron mayor diversidad según el índice inverso de Simpson. El subgrupo más abundante según el estimador de Chao1, fue el Est6sTx. La distribución de las unidades taxonómicas operacionales fue uniforme según el índice de Pielou (J) (cuadro 5).

**Figura 2:**
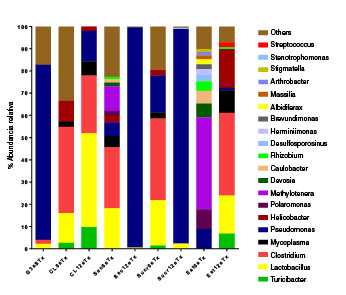
Composición de la microbiota obtenida de los sólidos del intestino delgado de ratones CD1 tratados o no con edulcorantes durante 6 y 12 semanas

En el grupo de ratones sometidos a consumo de edulcorante durante 6 semanas, se registraron diversos géneros. En el grupo CL6sTx, la abundancia relativa más alta correspondió a *Clostridium, Lactobacillus, Helicobacter, Turicibacter* y *Mycoplasma.* En los subgrupos Sac6sTx y Sucr6sTx, hubo un mayor porcentaje de abundancia relativa de *Clostridium* y de *Lactobacillus.* En el subgrupo Est6sTx, hubo una mayor abundancia de *Methylotenera,* con presencia de otros géneros como *Devosia, Caulobacter, Rhizobium, Desulfosporosinus* y *Albidifereax,* entre otros (cuadro 6) (figura 2). En el G12sTx, el subgrupo CL12sTx mostró mayor abundancia de *Lactobacillus* con presencia de otros géneros como *Clostridium, Turicibacter, Mycoplasma* y *Helicobacter.* Hubo una disminución significativa en la diversidad bacteriana de las muestras de Sac12sTx, con una mayor abundancia relativa del género *Pseudomonas,* en tanto que, en el subgrupo Sucr12sTx, hubo presencia de *Pseudomonas* y *Lactobacillus* (cuadro 6). Por otra parte, en el grupo Est12sTx, la mayor abundancia correspondió a *Clostridium, Helicobacter, Lactobacillus, Mycoplasma, Turicibacter, Streptococcus* y *Pseudomonas.* Las frecuencias relativas de los géneros bacterianos predominantes basadas en las secuencias del gen ARN ribosómico 16S (16S rRNA), se presentan en el cuadro 6 y la figura 2.

Los datos obtenidos significan las medias de la secuenciación masiva de las regiones V6-V8 del gen 16S rRNA. Clasificación taxonómica con abundancia relativa a nivel de género de la lectura total de las secuencias. El análisis se hizo en MOTHUR utilizando la alineación de referencia bacteriana de Silva. Grupo de 3 semanas sin tratamiento (G3sSTx); grupos con 6 semanas de tratamiento (G6sTx): control (CL6sTx), sacarosa (Sac6sTx), sucralosa (Sucr6sTx) y estevia (Est6sTx); grupos con 12 semanas de tratamiento (G12sTx): control (CL12sTx), sacarosa (Sac12sTx), sucralosa (Sucr12sTx) y estevia (Est12sTx)

**Figura 3:**
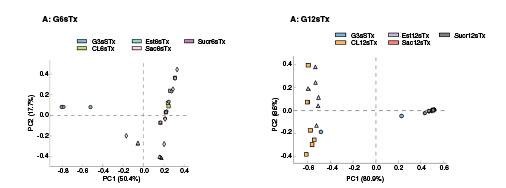
Gráfico del análisis coordinado principal (plot ACoP) de comunidades bacterianas presentes en el contenido sólido del intestino delgado de ratones CD1 que consumieron edulcorantes durante 6 semanas (A: G6sTx) y 12 semanas (B: G12sTx). G3sSTx. Grupos con 6 semanas de tratamiento (G6sTx): control (CL6sTx), sacarosa (Sac6sTx), sucralosa (Sucr6sTx) y estevia (Est6sTx); grupos con 12 semanas de tratamiento (G12sTx): control (CL12sTx), sacarosa (Sac12sTx), sucralosa (Sucr12sTx) y estevia (Est12sTx)

Además, se analizaron los componentes principales para todas las lecturas de las secuencias del gen 16S rRNA según el tiempo de tratamiento para detectar diferencias en la composición bacteriana en las muestras. En el grupo de tratamiento G6sTx, los dos componentes principales explicaron el 70 % de la varianza de la diversidad beta de las muestras. En la figura 3, se observa que los subgrupos CL6sTx, Sac6sTx y Sucr6sTx se agruparon de manera positiva para ambos componentes, en tanto que el subgrupo de Est6sTx se agrupó de manera negativa para el PC2, lo que puede explicar el 17,7 % de la varianza. Además, el G3sSTx se agrupó bajo valores negativos para el PC1, lo que explicaría el 50,4 % de la varianza. Estas diferencias indican una mayor heterogeneidad en G3sSTx y Est6sTx (figura 3).

En los grupos tratados durante 12 semanas, los dos componentes principales explicaron un 81,5 % de la varianza. Los subgrupos Sac12sTx y Sucr12sTx presentaron una menor diversidad, agrupándose positivamente en el PC1 y explicando el 80,9 % de la varianza. El subgrupo CL12sTx puntuó negativamente para ambos componentes y el subgrupo Est6sTx se ubicó positivamente en el PC2, el cual explica únicamente el 8,6 % de la varianza (figura 3).

## Discusión

### Consumo de alimento, agua y edulcorante, aporte energético, peso corporal e IMC, según cada edulcorante

*Sacarosa.* En el G6sTx, los ratones disminuyeron su consumo de alimento y agua y, por lo tanto, el aporte calórico, con un incremento sustancial del consumo de sacarosa y sin cambios en el IMC. Este comportamiento podría deberse a que el gran consumo de sacarosa pudo ocasionarles sensación de saciedad, lo que resultó en la moderación del consumo de alimento. Ello coincide con un estudio previo en el que se señaló cómo el consumo crónico de alimentos dulces con sacarosa podría inducir comportamientos adictivos y desembocar en una mayor ingestión de alimentos dulces y menor consumo de alimento ^36^. Tales conductas adictivas se han visto también al administrar soluciones endulzadas con sacarosa a ratas, con la consecuente disminución en el consumo de alimento ^37^. En otro estudio en el que los ratones consumieron glucosa, su sensibilidad a la insulina aumentó y el porcentaje de grasa corporal se elevó ^38^, aunque no hubo modificaciones en el IMC.

En este sentido, todavía hay controversia sobre si las dietas que contienen hidratos de carbono, como sacarosa, maltodextrinas y almidón, inducen cambios metabólicos ^39^. En otro estudio en ratas expuestas a sacarosa, se observaron reacciones fisiológicas y conductuales similares a las provocadas por el abuso de drogas como la cocaína o las anfetaminas ^40^, lo que explicaría el gran consumo de sacarosa en este estudio. Lo que es un hecho es que la exposición repetida a la sacarosa ocasiona tolerancia a sus efectos, lo cual se ha comprobado en animales que desarrollaron una reacción compensatoria caracterizada por la hiperactividad asociada con su consumo ^41^.

*Sucralosa.* En el subgrupo Sucr6sTx, los ratones no modificaron la cantidad de alimento consumido en comparación con el subgrupo de control, sin embargo, disminuyeron su consumo de agua. Los ratones consumieron mayor cantidad de sucralosa que de estevia (22,7 mg/ml de sucralosa *Vs.* 14,31 mg/ml de estevia), con escaso aporte energético, pero sin cambios en el IMC (cuadro 2). En el G12sTx, el consumo de alimento y agua y el aporte energético disminuyeron y hubo un mayor consumo de edulcorante (28,47 mg/ml) en comparación con el G6sTx (22,77 mg/mL), aunque sin cambios en el IMC (cuadro 2).

Los estudios sobre la sucralosa han suscitado controversias. En uno con ratas macho, se encontró que el consumo de sucralosa durante 12 semanas aumentó la expresión de la P-glucoproteína y el citocromo P450, lo que sugiere que el cuerpo podría estar tratando a la sucralosa como una toxina que requiere ser eliminada ^42^. La sucralosa es un edulcorante artificial intenso cuya dulzura se correlaciona con fuertes enlaces de hidrógeno, agua y azúcar ^39^, lo cual genera preferencia por su consumo, pero con la ventaja de que no aporta calorías a diferencia de la sacarosa. A pesar de su inocuidad, hasta ahora continúa la controversia frente a su efecto sobre el IMC.

*Estevia.* El consumo de estevia durante 6 semanas (Est6sTx) disminuyó el consumo de alimento y no produjo cambios en el consumo de agua, comparado con el grupo de control. Se consumieron 14,31 mg/semana de estevia, proporción mucho menor que la del consumo de sacarosa y sucralosa (cuadro 2). El aporte energético fue el más bajo de los tres grupos y no hubo cambios en el IMC como sucedió con la sucralosa. En el subgrupo Est12sTx, no se apreciaron cambios en el consumo de alimento o agua, en el aporte energético ni el IMC, y los ratones elevaron el consumo de estevia comparado con el subgrupo tratado por 6 semanas (Est6sTx). Se ha reportado que el consumo de estevia en el desayuno reduce el consumo de alimento durante el resto del día en comparación con los alimentados con sacarosa ^43^. En este estudio el edulcorante se administró en la mañana, lo que podría explicar este comportamiento.

En cuanto al IMC y su relación con el consumo de edulcorantes no nutritivos, esta sigue suscitando controversia, aunque se sabe que es multifactorial y depende del sexo, la edad, el tipo de dieta, el estilo de vida e, incluso, la raza ^3^. Los resultados del presente estudio coinciden con lo reportado por Figlewicz, *et al.* y Dhingra, *et al.,* autores que concluyeron que el consumo de sucralosa y estevia no aumentó el peso corporal ni el IMC en ratas ^44^^,^^45^, aunque también subrayan las diferencias debidas a la especie o sexo.

En este sentido, el contenido de la dieta actúa de forma distinta en cada individuo, como se resalta en el estudio de Nishikawa, *et al.* Los autores compararon seis cepas diferentes de roedores que, a pesar de recibir la misma dieta, se comportaron de forma diferente, pues cada una reguló la cantidad de alimento consumido ^46^, situación que también se apreció en nuestro estudio, especialmente con el consumo de sacarosa. Por otro lado, el gusto influye en la predilección por uno u otro nutriente. En el estudio de South, *et al.,* se observó que cada cepa de ratones prefirió un tipo particular de alimento que, en este caso, fue la sacarosa frente a la sucralosa y la estevia ^47^. Se han descrito, además, cepas resistentes a la dieta, ya que no ganan peso aun cuando se les brindan dietas elevadas en grasa o hidratos de carbono ^48^.

En el presente estudio, el IMC no se modificó en ningún subgrupo, ni siquiera cuando los animales tuvieron un consumo prolongado de edulcorantes. En el caso de la sucralosa y la estevia, esto puede explicarse por su bajo índice calórico, pero la sacarosa, a pesar de su alto índice glucémico, ocasionó una disminución en el consumo de alimento, lo que permitió que el IMC no se incrementara. Asimismo, resulta benéfico reducir la exposición al sabor dulce en la dieta, ya que los edulcorantes artificiales, aunque reducen el consumo de calorías, fomentan la ansiedad y la dependencia ^49^. Otro factor que explica este fenómeno es el hecho de que los edulcorantes activan señales intestinales y gástricas que ocasionan un efecto sinérgico sobre la saciedad, además de modular el consumo de alimento ^2^^,^^37^.

Se considera que el aumento de peso en los individuos es de origen multifactorial (genético, ambiental, conductual, etc.). Las dietas ricas en glucosa y calorías influyen en el apetito de los animales, ya que disminuyen el consumo de alimento ^50^, situación que se observó con el consumo de sacarosa en el presente estudio. Así pues, la dieta por sí sola no produce un aumento significativo de peso. La controversia sobre los efectos del consumo de edulcorantes bajos en calorías en la salud continúa, pues se ha asociado positivamente con la ganancia de peso y la diabetes, pero también con un IMC bajo y pérdida de peso, además de casos sin relación con el peso corporal y ciertos parámetros metabólicos ^51^.

*Consumo de edulcorante y glucemia.* La glucemia no se modificó con el consumo de edulcorantes. Aunque la sacarosa tiene un mayor aporte calórico (4 kcal/g), los niveles de glucemia no fueron estadísticamente diferentes a los de los ratones que consumieron sucralosa (menos de 2 kcal/g). Estos resultados coinciden con los obtenidos recientemente por Grotz, *et al.,* quienes demostraron que el consumo de sucralosa y estevia no aumentaban la glucemia ^52^.

### Relación con la microbiota de los marcadores inmunológicos en las placas de Peyer y la lámina propia

En el intestino delgado residen aproximadamente 104 unidades formadoras de colonias por gramo (UFC/g) de contenido intestinal en la parte superior y 108 UFC/g en la región distal del íleon, cifra que aumenta (1014 UFC/g) en el intestino grueso ^53^. Hay diversos factores que influyen en la composición de las comunidades bacterianas, como las modificaciones en los gradientes químicos o en la disposición de nutrientes, y la situación inmunitaria del huésped ^54^. Sucede igual en la homeostasis de las comunidades bacterianas, especialmente en el estado nutricional del individuo ^55^. Cuando este equilibrio se pierde, se producen modificaciones en el tipo de especies y su abundancia ^56^, y se altera su comunicación con las vías inmunitaria y nerviosa. Por ejemplo, se ha descrito, que interfieren en la elaboración de moléculas de señalización y el reconocimiento de compuestos bacterianos por parte de las células inmunitarias del epitelio y de la lámina propia ^57^. El estudio de la microbiota del intestino delgado tiene gran importancia para el funcionamiento endocrino y del tejido linfoide asociado al intestino, pues en él se lleva a cabo cerca del 90 % de la extracción de energía proveniente de la dieta ^56^; además, la absorción de nutrientes depende del buen funcionamiento e integridad de la mucosa, incluida la microbiota ^58^. La mayoría de las investigaciones en este campo se enfocan en el estudio de la microbiota derivada de heces fecales del intestino grueso ^59^, por lo que es la que cuenta con la descripción más completa hasta el momento.

*Sacarosa.* Al comparar los parámetros inmunitarios por compartimiento, se pudo observar que la sacarosa tuvo un efecto importante cuando su consumo fue prolongado (12 semanas), ya que elevó el porcentaje de linfocitos CD19+ y disminuyó el de las células IgA+ en las placas de Peyer, debido, quizá, a la falta de cambio del isotipo de linfocitos a células plasmáticas al reducirse la secreción de TGF-beta, principal estimulador de este proceso.

En la lámina propia, no se modificó el porcentaje de linfocitos CD19+ y las células IgA+ se incrementaron quizá por el aumento en la migración desde las placas de Peyer y el cambio de isotipo por la estimulación del TGF-beta elevado. En ambos compartimientos se incrementaron las interleucinas IL-12 e IL-17 (cuadro 4), lo que permite concluir que la sacarosa podría actuar como estimulador de los linfocitos B vírgenes para transformarlos en células plasmáticas productoras de IgA+.

La diversidad bacteriana fue mayor en el grupo G6sTx que en el G12sTx (figura 2) (cuadros 5 y 6). La gran diversidad bacteriana observada a las 6 semanas de tratamiento pudo deberse a la estimulación de la sacarosa en las placas de Peyer, con la consecuente elevación de la IL-12 y la IL-17 ^60^. La estimulación del compartimiento inductor (placa de Peyer) podría explicarse por el incremento del porcentaje de células IgA+ y de las citocinas TGF-beta, IL-12 e IL-17 en la lámina propia. Por el contrario, la diversidad bacteriana se redujo con el consumo de sacarosa durante 12 semanas, ya que solo se encontraron proteobacterias del género *Pseudomonas.* Esta reducción en las comunidades bacterianas puede explicarse también por la reducción en el porcentaje de células IgA+, lo que ocasiona una transición incompleta a una microbiota madura y, por lo tanto, mayor propensión a las lesiones intestinales ^61^. La producción elevada de citocinas IL-12 e IL-17 podría ser la consecuencia de una reacción protectora para mantener la homeostasis y la integridad de la barrera intestinal y, quizá, un intento de estimular a la población bacteriana y revertir su reducción.

Se puede inferir, entonces, que el tiempo prolongado de consumo de sacarosa modificaría el comportamiento de los linfocitos de las placas de Peyer y la lámina propia, y alteraría a largo plazo la composición microbiana, ocasionando una disbiosis. Se ha reportado que los edulcorantes artificiales ocasionan disbiosis microbiana y efectos metabólicos adversos en los roedores ^62^ y, a pesar de que la sacarosa no es un edulcorante artificial, podría tener un efecto similar. Por otro lado, los niveles elevados de IL-12 e IL-17 pueden ser una reacción protectora para mantener la homeostasis y la integridad de la barrera intestinal ^60^ (figura 2) (cuadro 6).

Este efecto coincide con un reporte que indica que las dietas ricas en hidratos de carbono simples, como la sacarosa, reducen notablemente el ecosistema bacteriano ^63^. Si se toma en cuenta que los azúcares y edulcorantes se absorben activamente en el intestino delgado a través de transportadores de azúcar, y que únicamente entre el 5 y el 30 % de estos llega al intestino grueso ^64^, resulta congruente que exista mayor interacción en la mucosa y la luz del intestino delgado que en el intestino grueso. Estos azúcares son sustratos muy utilizados que enriquecen a los microbios del intestino delgado en comparación con los del intestino grueso ^65^. Otro ejemplo es el de los hidratos de carbono, como la fructosa, los alcoholes de azúcar y algunos edulcorantes, entre ellos la sucralosa, que son poco o pasivamente absorbidos en el intestino delgado, por lo que la variación de los microbios encontrados *depende de la* variación de los azúcares o edulcorantes presentes allí ^64^.

*Sucralosa.* A las 6 semanas de tratamiento (G6sTx), los linfocitos CD19+ de las placas de Peyer aumentaron significativamente, al igual que las células IgA+, el TGF-beta, la IL-12 y la IL-17. En la lámina propia, en cambio, los linfocitos CD19+ disminuyeron, con un incremento de las células IgA+, el TGF-beta, la IL12 y la IL-17. Estas últimas se incrementaron en ambos compartimientos, quizá como consecuencia de su interacción con la microbiota modificada constituida por proteobacterias, firmicutes y tenericutes, y géneros diversos como *Helicobacter, Pseudomonas, Lactobacillus, Clostridium, Turicibacter* y *Mycoplasma.*

En el grupo G12sTx, en cambio, se mantuvo en las placas de Peyer el incremento de los linfocitos CD19+ y la secreción de IL-17, pero se redujo la cantidad de células IgA+ y la secreción de TGF-beta e IL-12. En la lámina propia, se observó incremento de CD19+ e IgA+, quizá por el aumento en su migración desde la placa de Peyer y su estimulación por parte del TGF-beta elevado. Como se puede apreciar, a las 12 semanas los cambios en los parámetros inmunitarios en ambos compartimientos podrían indicar que el consumo crónico de sucralosa sí afectó la inmunidad de la mucosa del intestino delgado.

La elevación de los linfocitos BCD19+ y la IL-17 en las placas de Peyer podría ser un mecanismo compensatorio, con una marcada disminución de IgA+, lo cual indicaría que no hubo una activación de los linfocitos BCD19+ hacia células plasmáticas productoras de IgA+, o cambio de isotipo, o que las células migraron aceleradamente hacia la lámina prima, donde su número aumentó. El incremento de TGF-beta, IL-12 e IL-17 puede explicarse por una mayora migración de los linfocitos BCD19+ de las placas de Peyer hacia la lámina propia, con estimulación *in situ* de los linfocitos BCD19+ para la activación y el cambio de isotipo, y con la consecuente estimulación en la secreción de IL-12 e IL-17 en la lámina propia.

Esto puede deberse a la reducción de la diversidad bacteriana que ocasionó poca estimulación de la mucosa intestinal, ya que únicamente se encontraron dos clases de organismos, proteobacterias y firmicutes, y dos géneros, *Pseudomonas* y *Lactobacillus.* Se puede inferir, entonces, que cuanto más prolongado es el tiempo de consumo de la sucralosa, mayor es el daño a la diversidad de la microbiota del intestino delgado y, por ende, mayor la modificación de la población de linfocitos BCD19+ y células IgA+. El tiempo prolongado de consumo sí afecta la composición bacteriana.

No obstante, los resultados aún son contradictorios, ya que ahora se pone en duda que la sucralosa no se metabolice y se excrete sin cambios. Se ha descrito en ratas que la sucralosa se metaboliza en compuestos que son menos polares y, por lo tanto, más lipofílicos que el compuesto original. Se encontró que la sucralosa se retuvo en el tejido adiposo dos semanas después del cese de la ingestión ^66^. Por lo tanto, el concepto de su inocuidad, no absorción y los posibles efectos crónicos del consumo habitual, se han cuestionado. Los resultados de varios estudios recientes plantean una serie de problemas con respecto a la seguridad potencial de la ingestión crónica de la sucralosa en humanos ^67^.

Otro hallazgo sugiere que esta tiene mayor tendencia a unirse con bolsas hidrofóbicas en las superficies de las proteínas, lo cual podría provocar su interacción débil con ellas y la desestabilización de sus estructuras ^68^. Este mecanismo podría influir en la desestabilización de las membranas celulares de algunos tipos de bacterias de la microbiota intestinal y, con ello, disminuir la población bacteriana, lo que ocurrió en este estudio. Se ha documentado que dicho desequilibrio y la reducción en la composición de la microbiota intestinal, son parcialmente responsables de enfermedades como alergias, cáncer gástrico, enfermedad de Crohn, obesidad y enfermedad inflamatoria intestinal ^67^. Sin embargo, no se ha reportado una relación directa entre el consumo de sucralosa y estas enfermedades, y se reconoce, más bien, como uno de los factores causales, especialmente en la enfermedad inflamatoria intestinal ^69^.

En la obesidad sí se ha demostrado que la sucralosa altera la relación huésped-microbio en el intestino ^70^, pero sigue siendo un hecho que los estudios todavía son contradictorios en este aspecto. Por ejemplo, en el estudio de Abou-Donia, *et al.,* en ratas macho suplementadas durante 12 semanas con Splenda, edulcorante que contiene sucralosa, se reportó una reducción de las bifidobacterias, lactobacilos, *Bacteroides,* clostridios y las bacterias aeróbicas totales, con pérdidas de hasta 79,7 % en los lactobacilos. Los autores concluyeron que la sucralosa administrada como Splenda reduce el número de bacterias intestinales autóctonas y promueve una mayor supresión de anaerobios usualmente benéficos (lactobacilos y bifidobacterias), con menos inhibición para bacterias perjudiciales (enterobacterias) ^70^.

En el presente estudio, la composición bacteriana en el G6sTx se redujo y modificó, y solo se registraron los géneros *Helicobacter, Pseudomonas, Lactobacillus, Clostridium, Turicibacter,* y *Mycoplasma,* en tanto que, en el G12sTx, se evidenció una pérdida casi total de las comunidades bacterianas, con presencia únicamente de *Pseudomonas* y *Lactobacillus.* La controversia persiste, ya que según Brusick, *et al.,* los resultados en cuanto a la modificación de la microbiota con el consumo de sucralosa no son concluyentes ^71^, pues se asevera que esta también ocurre como resultado de una variación biológica normal ^72^. Por último, se plantea que la perturbación de *Bacteroides* por consumo de sucralosa aumenta la posibilidad de afectar la estabilidad de todo el ecosistema bacteriano en el tubo digestivo, ya que esta tiene un papel crítico en la estabilidad y la resistencia de la colonización intestinal ^66^.

*Estevia.* El comportamiento de los grupos tratados con estevia fue completamente diferente al de los tratados con sacarosa y sucralosa. En el G6sTx, la estevia se comportó como un estimulador de las placas de Peyer, ya que se incrementó el porcentaje de linfocitos BCD19+, IgA+, TGF-beta e IL-17, lo cual también explicaría la gran diversidad bacteriana que se encontró en este grupo (figura 2). La IL-12 disminuyó significativamente, quizá por no reconocer a la estevia como un patógeno, sino porque permite una adecuada relación simbiótica entre la microbiota y la mucosa del intestino delgado. Ahora bien, en la lámina propia se incrementaron los linfocitos BCD19+ y la IL-17, pero disminuyeron las células IgA+, tal vez debido a que las células de la placa de Peyer no migraron en abundancia, ya que el estímulo no fue reconocido como nocivo, lo que también redujo la secreción de TGF-beta e IL-12.

La diversidad bacteriana incluyó, principalmente, proteobacterias, firmicutes y actinobacterias, con 13 géneros diferentes, entre ellos: *Devosia, Caulobacter, Rhizobium, Methylotenera, Polaromonas, Brevundimonas, Albidiferax, Masilia, Stigmatella, Pseudomonas, Desulfosporosinus, Mycoplasma* y *Athrobacter* (figura 3). El mismo comportamiento se pudo apreciar en el G12sTx (cuadro 6), aunque con modificaciones en la diversidad bacteriana con respecto al G6sTx, ya que continuaron apreciándose las proteobacterias y firmicutes, pero no así las actinobacterias; en su lugar, aparecieron tenericutes y siete géneros: *Helicobacter, Pseudomonas, Lactobacillus, Clostridium, Turicibacter, Streptococcus* y *Mycoplasma.*

Entre las propiedades de la estevia estudiadas hasta el momento, se encuentran su actividad antidiabética, anticariogénica, antioxidante, hipotensiva y antihipertensiva, antiinflamatoria y antitumoral. Como parte de su actividad antimicrobiana, *Estevia rebaudiana* inhibe el crecimiento de ciertas bacterias patógenas, por lo cual se utiliza para tratamiento de heridas, llagas y enfermedades de encías ^73^. Su actividad bactericida se ha estudiado en extractos acuosos que resultaron efectivos contra *Bacillus subtilis, Staphylococcus aureus, Escherichia coli* y su variedad enterohemorrágica, y *Salmonella typhimurium*^74^.

La estevia aparentemente no afecta la microbiota intestinal endémica, lo que se explicaría porque favorece su abundante proliferación y no daña o compite por los sustratos de las bacterias, favoreciendo su abundancia y diversidad. Asimismo, es un hecho que los fructanos derivados de la planta mejoran el crecimiento de cepas microbianas seleccionadas como las bifidobacterias y los lactobacilos, importantes para la función intestinal ^75^ . Los organismos de *Bacteroides* son abundantes en el tubo digestivo y pueden hidrolizar eficientemente el esteviósido y el rebaudiósido A a esteviol, lo que no sucede con los lactobacilos, las bifidobacterias, *Clostridium,* y las especies de coliformes y enterococos, ya que no utilizan glucósidos de esteviol como sustrato ^62^.

En el presente estudio, no se registró la presencia de *Bacteroides* en ninguno de los tres grupos de estudio, por lo tanto, se puede aseverar que es una especie propia del colon que no es abundante en intestino delgado. Lo cierto es que la estevia aumenta el índice de diversidad de Shannon en el colon debido a su efecto sobre la uniformidad de la comunidad de microbiomas, más que por un impacto en la riqueza de unidades taxonómicas operacionales ^75^.

Se encontraron, en el presente estudio, proteobacterias del género *Methylobacteria,* las cuales se utilizan como acelerador del crecimiento y están presentes de forma habitual en las hojas de la planta de *E. rebaudiana*^76^ . La presencia de esta bacteria no se detectó en el grupo de sucralosa, pero sí en el de sacarosa (G6sTx). Además, se ha observado en muestras de heces fecales de ratones de la misma edad y condición una gran proporción de firmicutes y actinobacterias y, en menor proporción, proteobacterias y tenericutes ^77^. A pesar de que las muestras en este estudio proceden del contenido sólido del intestino delgado, la presencia de firmicutes y la dominancia de proteobacterias fueron constantes, como se ha reportado en otros estudios ^78^. La elevada prevalencia de proteobacterias se ha interpretado como un posible marcador de disbiosis y, por lo tanto, de riesgo de enfermedad ^79^.

Lo que se ha podido comprobar, en general, es que el uso de edulcorantes no calóricos puede alterar la proliferación de las proteobacterias en el intestino, particularmente en el colon, comportamiento que en este estudio también se observó en el intestino delgado ^80^. Otro ejemplo de esta disbiósis es el incremento de las betaproteobacterias, que se relaciona con alteraciones metabólicas como la diabetes mellitus de tipo 2 ^81^. Lo dicho deja claro el hecho de que el tipo de dieta modifica la composición de la microbiota en ambas porciones del intestino.

El consumo de agua y alimento, y el aporte energético disminuyeron con el consumo de sacarosa a las 12 semanas, sin cambios en el IMC ni la glucemia. El edulcorante más consumido por los ratones fue la sacarosa. Después de 12 semanas de consumo de sacarosa y sucralosa, se incrementaron en las placas de Peyer los porcentajes de CD19+ e IL-17, con disminución de lgA+ y de TGF-b, así como de la diversidad bacteriana, especialmente con el consumo de sucralosa. Por el contrario, la estevia mejoró el porcentaje de linfocitos BCD19+, lgA+, TGF-b, lL-12 e lL-17, y mantuvo niveles favorables de diversidad bacteriana. La estevia interactuó de forma saludable con la mucosa intestinal, comparada con la sacarosa y la sucralosa.
